# Supplementary material for: Perfect secrecy cryptography via mixing of chaotic waves in irreversible time-varying silicon chips
Source: Nat Commun. 2019 Dec 20;10:5827. doi: 10.1038/s41467-019-13740-y (PMC6925125; doi:10.1038/s41467-019-13740-y)
Supplement: Supplementary file 1 — Supplementary Information [file 41467_2019_13740_MOESM1_ESM.pdf]

Perfect secrecy cryptography via mixing of chaotic waves in irreversible time varying silicon chips

SI

A. Di Falco, V. Mazzone, A. Cruz & A. Fratalocchi

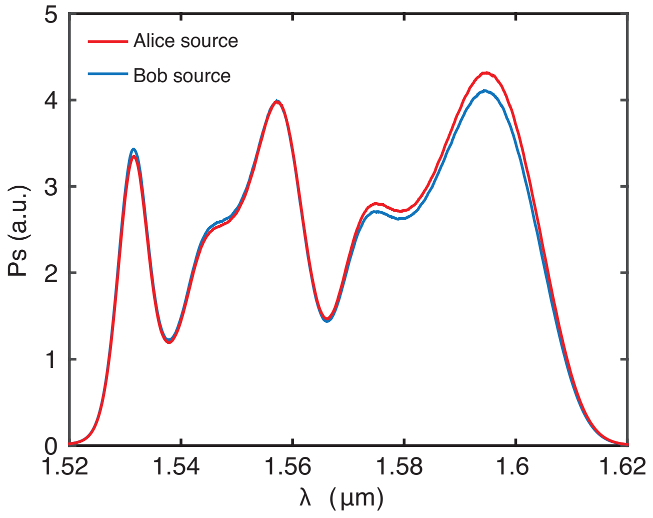

Supplementary Figure 1. **Power density source spectra** Power density spectrum $P_S(\omega)$ of the source used by Alice (red line) and Bob (blue line), measured with the spectrometer used by each user.

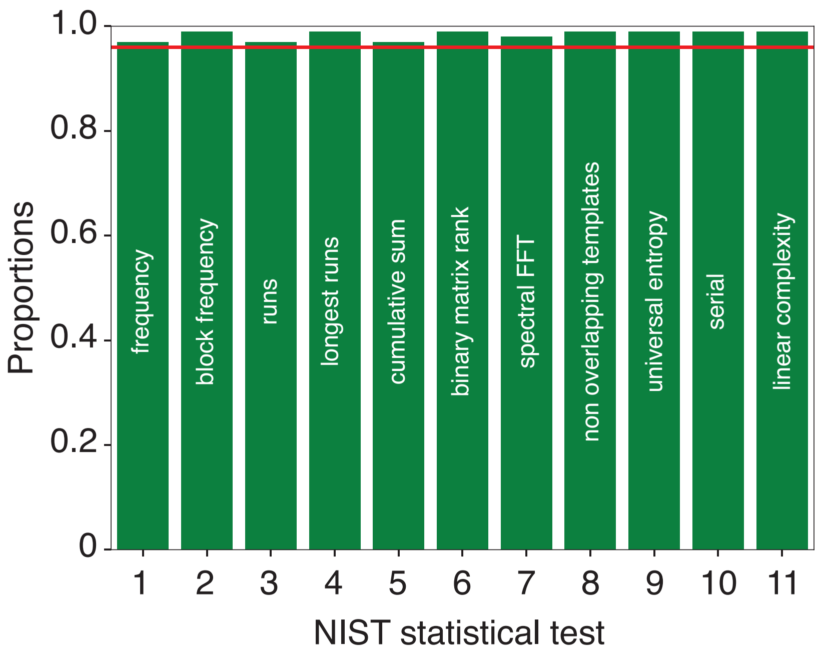

Supplementary Figure 2. **NIST statistical tests on generated keys** Proportions results on NIST statistical tests for cryptography applications from a statistic population of 1000 keys each of 1000 bits. The red line indicates the minimum threshold to pass each test.

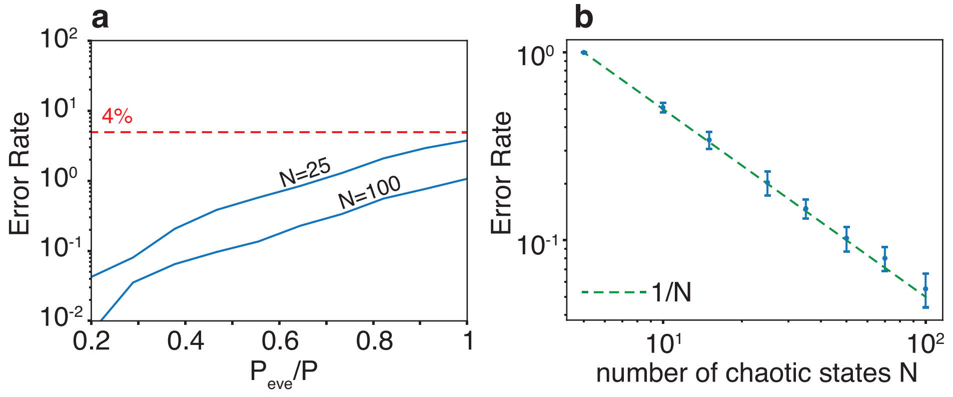

Supplementary Figure 3. **Security analysis in the case of active attacks**. Error rate introduced in the communication by active intercept-resend attacks as a function of (a) the state changing probability of Eve and (b), the number of chaotic states N in each chip. The simulations are performed from the analysis of 10^7 sequences of different states. Each element of the sequence for Alice and Bob is an integer random number $n \in [0, N]$ and $n' \in [0, N]$, respectively, representing the chaotic wavepacket generated by the input conditions n and n' in Alice and Bob chips. We assume each chip containing N independent and uncorrelated chaotic states. The generation of a new state in the sequence happens with probability P, identical for Alice and Bob. The action of Eve is simulated at each step by the substitution of a state with a new one from another chip, introducing a communication error between the repeated sequences of Alice and Bob. Eve's decision to change the state occurs with probability P_{eve}.

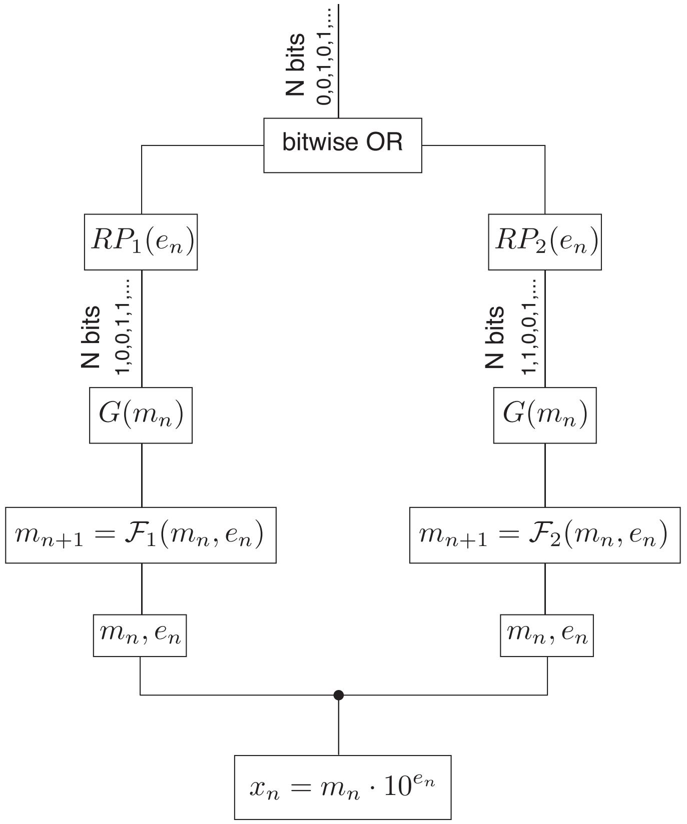

Supplementary Figure 4. **Block scheme of the multi-bit AHB transform**.

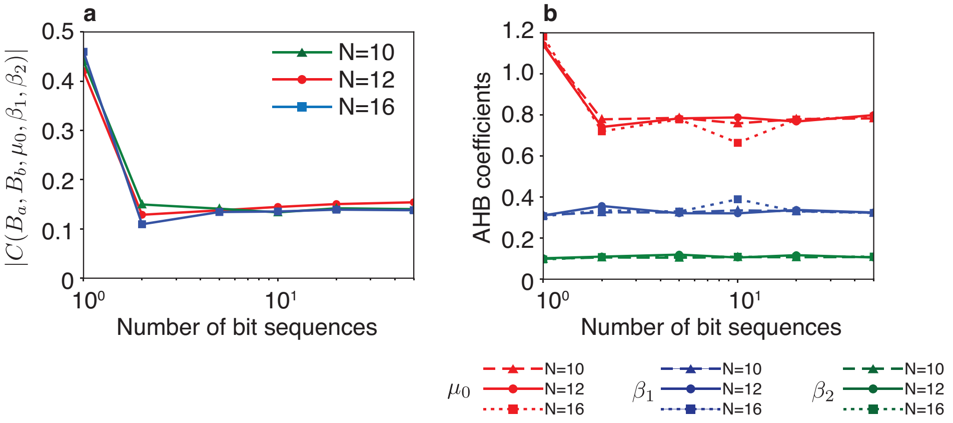

Supplementary Figure 5. **Training of the nonlinear AHB transform for $\sigma_{AB} = 8\%$** (a) Norm of the cost function $|C|$ and (b) optimized AHB coefficients (μ_0, β_1, β_2) versus the number of bit sequences N_{ch} composing the training dataset.

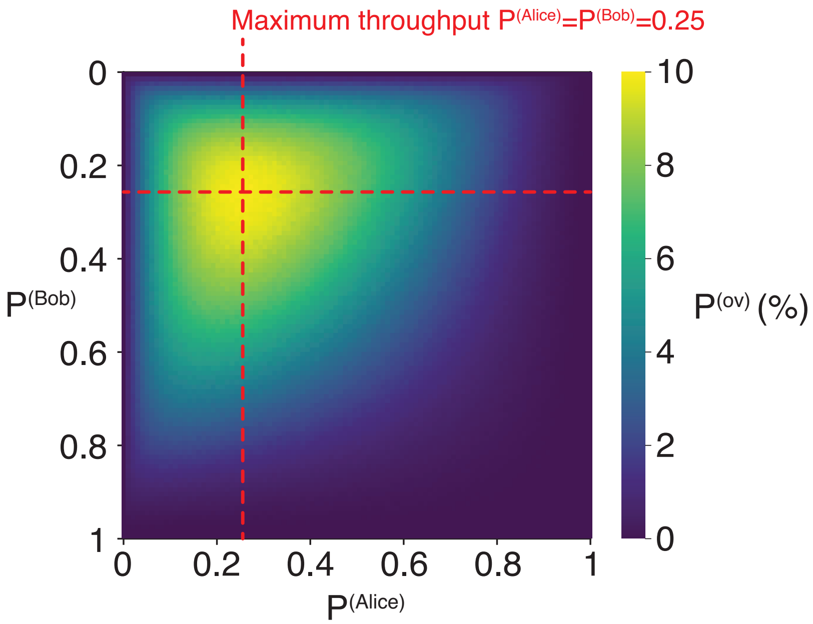

Supplementary Figure 6. **Maximum key generation rate** Probability $P^{(ov)}$ of observing repeated states at the same position in Alice and Bob's sequences as a function of the users probabilities $P^{(Alice)}$ and $P^{(Bob)}$ to change state after each repeated communication. The calculations are performed on a statistical sample of 1000 sequences, each of 100000 states.

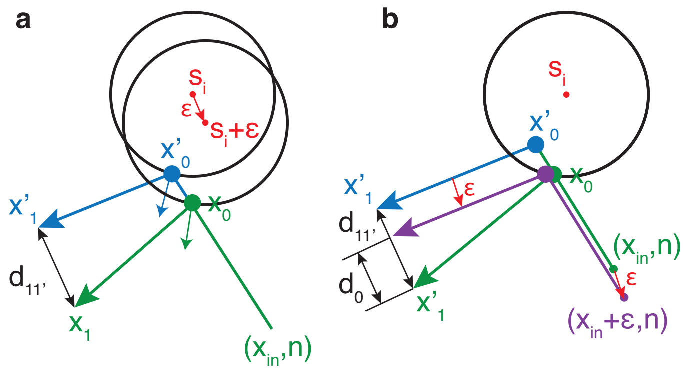

Supplementary Figure 7. **Dynamics of light rays in spatially displaced scatterers** (a) Geometrical calculation of reflected trajectories of an identical input condition impinging two scatterers displaced by ϵ. (b) Equivalent calculation in a configuration in which the scatter is held fixed.

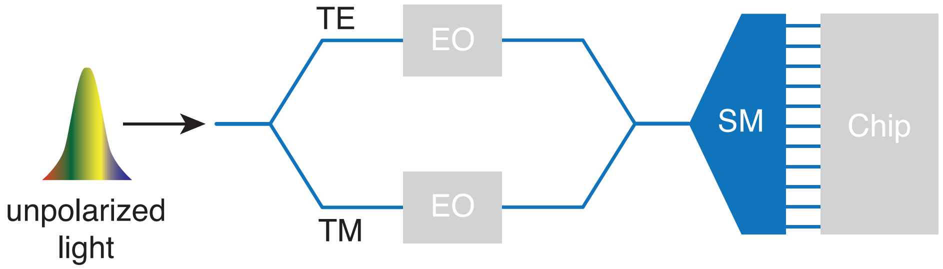

Supplementary Figure 8. **Integrated realization with on-chip light couplings from end-to-end** EO: electro-optic modulator, SM: switch matrix.

SUPPLEMENTARY NOTE I

We generated chaotic states $A_n(t)$ and $B_{n'}(t)$ possessing $m = 1, ..., M$ different frequencies $\omega_m = \frac{2\pi}{\lambda_m}$ and $\lambda_m = m$, acquiring time sequences with random amplitudes $a_{nm}, b_{nm} \in [0, 1]$, phases $\phi_{nm}, \psi_{n'm} \in [0, 2\pi]$.
Each generated state $A_n(t)$ and $B_{n'}(t)$ is then converted into a bit sequence of length $L = 10000$ bits by the adaptive high boost (AHB) technique. The AHB transformation initially applies a transform Δ, such as, e.g., a convolution kernel, to the data sequence to be converted, and then uses a Gabor transform G to generate a binary sequence. For a given sequence of numbers Y_l $(l = 1, ..., L)$, the AHB is compactly represented as follows:

$$\text{AHB}\{Y_l\} = G\{\Delta(Y_l)\}, \quad G\{X_l\} = \begin{cases} 0, X_l < th \\ 1, X_l \geq th \end{cases}, \quad (1)$$

In our analysis we used a differential convolution filter $\Delta(Y_l) = \Delta \otimes Y_l = [1, -1] \otimes Y_l$, with $\otimes$ a discrete convolution, and a Gabor transform with threshold $th = 0$.
The average uncertainty per bit is evaluated as follows. After chaotic states are converted into binary sequences, we calculated the normalized hamming distance d_{ab} among two binary sequences a and b as follows:

$$d_{ab} = \frac{1}{L} \sum_{l=1}^{L} |a_l - b_l|. \quad (2)$$

This value furnishes the probability with which, on average, one bit is different in the two sequences. We then converted the resulting value to Shannon information entropy $H = -d_{ab} \log_2 d_{ab} - (1 - d_{ab}) \log_2(1 - d_{ab})$. We evaluated results from a statistics of 10000 samples.

SUPPLEMENTARY NOTE II

In the case of an active attack, Eve measures the data and decides to replace the state with another one from a different chip, with $E_1, ..., E_p$ states according to a probability P_{eve}. These attacks are particularly dangerous if they allow the enemy to gain information on the key without the users being aware of that.
The security of the protocol of Fig. 1 of the main text arises from the fact that, when active eavesdropping happens, the users measure uncorrelated states. If Alice and Bob send out A_i and Bob B_j, respectively, and Eve replace these states with E_x, Alice measures $E_x \oplus B_j$ and Bob $A_i \oplus E_x$. These states are not correlated: in the best ideal scenario the fake state E_x comes from a chip with a chaotic structure that is exponentially different from the one used by Alice and Bob (point 4 of the communication scheme). If Eve replaces one state, a user measures the correct state $A_i \oplus B_j$, while the other measures an uncorrelated state, either $E_x \oplus B_j$ or $A_i \oplus E_x$. The bidirectional communication of Fig. 1 of the main text can identify these error states and minimize their presence in the final key.
Supplementary Figure 3 illustrates the outcomes of active attacks for different probabilities of P_{eve}, normalized with respect to the probability chosen by the users P_{Alice}, P_{Bob} to change state after each communication step. We considered the best scenario for Eve, in which $P_{Alice} = P_{Bob} = P$. Figure 3a shows that even in the best scenario $P_{eve}/P = 1$ when Eve uses the same probability of Alice and Bob, the error rate introduced in the sequences measured by the users is small and below 4% with only $N = 25$ different chaotic waves. Supplementary Figure 3b shows that the error rate introduced by Eve is inversely proportional to the number of chaotic states N, and for a chip with a large number of states $N \gg 1$, the error rate introduced by this attack tends to zero.

SUPPLEMENTARY NOTE III

In an experimental implementation, the main statistical components of uncertainty is the fluctuation of the source Δ_S. The uncertainty contained in the reciprocal power density spectra of Alice and Bob, in fact, arises from the following uncorrelated components:

- Statistical fluctuations Δ_S of source spectrum.
- Noise thermal fluctuation Δ_C of the communication channel.
- Fluctuations Δ_A, Δ_B in the input conditions.
- Uncertainty Δ_M of the measurement apparatus.
- The coupling coefficient $\alpha(\omega)$: this is a systematic source of uncertainty (type B of NIST guidelines [1]). To evaluate $\alpha(\omega)$, the user must measure the absolute value of a spectrum and normalize it. To do so, the user must use another measurement instrument, which introduces an unknown coupling coefficient. This argument can be iterated at infinity, without the user knowing the first coefficient. It is the same problem of measuring the exact temperature of a body: one needs to know in advance the temperature of the thermometer, as it reads the equilibrium temperature between the coupled environments of the thermometer and the body.

Typical optical sources have statistical fluctuations in power amplitudes of the order of few percents. Just to make a specific example, the CL band tunable source T100S-HP from EXFO ensures an absolute wavelength accuracy better than ±20 pm and a power repeatability sweep-to-sweep better than ±0.05 dB, which implies differences as small as ±1% at every wavelength. The optics/optomechanics available today are stable to fluctuations below 10 nm, providing very little variations in

measured optical observable if compared to source fluctuations. Thermal fluctuations in the communication channel are also negligible and typically few orders of magnitude smaller than the source's amplitude. Measurement instruments available today have a dynamic range in the range of tens of dB and a spectral accuracy in the range of tens of picometers, thus providing very precise reading with standard deviation much smaller than sources amplitude variations. In an typical experimental realization, we are therefore in the situation where $\Delta_{A,B,C,M} \ll \Delta_S$. This is also a general case the users can always choose, as the source is controlled by the users at the time of the communication. In the limit of acceptable bit error rate (BER), Δ_S can always be increased if necessary by adding artificial time fluctuations.
The total standard uncertainty Δ when measuring a user spectrum P_{Bob} is then expressed by:

$$\Delta = \frac{\partial P_{Bob}}{\partial P_S}\Delta_S = |H_A^{(n)} \cdot H_B^{(n)} \cdot C \cdot \alpha|^2 \Delta_S \tag{3}$$

If Eve attempts a reconstruction of the spectrum P_{Bob}, she needs to perform at least three measurements:

a. The spectrum sent by Alice P_{EA}:

$$P_{EA}(\omega) = |S(\omega) \cdot H_A^{(n)}(\omega) \cdot C_A(\omega) \cdot \alpha(\omega)|^2, \tag{4}$$

with $C_A(\omega)$ the transfer function of the communication line connecting the Alice with Eve. The ideal scenario for Eve is to use an exact copy of the analyzer of Alice and Bob.

b. The spectrum sent by Bob P_{EA}:

$$P_{EB}(\omega) = |S(\omega) \cdot H_B^{(n)}(\omega) \cdot C_B(\omega) \cdot \alpha(\omega)|^2, \tag{5}$$

with $C_B(\omega)$ the transfer function of the channel between Bob with Eve.

c. The source spectrum $P_S = |S(\omega)\alpha(\omega)|^2$. This is essential: the use of chaotic uncorrelated spectra do not allow to estimate P_S from measurements a-b. The best choice for the attacker is to use a copy of the users spectrum analyzer. This minimizes any other statistical uncertainty due to, e.g., different sensors, resolution, and any different component.

Once measurements a-c are in place, Eve can attempt a reconstruction of P_{Bob} via $P_{EA} \cdot P_{EB}/P_S$. This operation returns:

$$\frac{P_{EA} \cdot P_{EB}}{P_S} = \langle P_{Bob} \rangle \frac{1}{|\alpha|^2} + \Delta' \tag{6}$$

with $C_A \cdot C_B = C$ and total total uncertainty Δ' evaluated from each independent measure performed by Eve:

$$\Delta' = \frac{1}{|\alpha^2|}\sqrt{\left(\frac{\partial P_{EA}}{\partial P_S}\right)^2 \frac{P_{EB}^2}{P_S^2} + \left(\frac{\partial P_{EB}}{\partial P_S}\right)^2 \frac{P_{EA}^2}{P_S^2} + \frac{P_{EA}^2 P_{EB}^2}{P_S^4}}\Delta_S = \sqrt{3}\frac{\Delta}{|\alpha|^2}. \tag{7}$$

Equations (6)-(7) show that in the ideal scenario the measurements of Eve contain at least one systematic multiplicative uncertainty, which Alice and Bob do not experience, and a statistical uncertainty that is at least $\sqrt{3}$ higher that the one of Bob and Alice. These results are the theoretical limit for an adversary with an infinite technological power. While nor the users neither the attacker know $\alpha(\omega)$, the statistical uncertainty Δ_S is a quantity that cannot be removed by the adversary as the source is possesses by the users during the communication.

SUPPLEMENTARY NOTE IV

Supplementary Figure 4d provides a block scheme of the multi-bit AHB transform. Each single point x_n in the acquired user's spectrum is first decomposed into its mantissa m_n and unsigned exponent e_n. The physical data generated (m_n, e_n) is then modified by two nonlinear filter functions $m_{n+1} = \mathcal{F}_{12}(m_n, e_n)$ N times, generating two independent data sequences that are then converted into binary by a Gabor transform with threshold $th = 0.5$.
The two generated sequences are randomly permuted consecutively in RP_1 and RP_2, by using a random seed equal to the exponent e_n, and finally merged together with a bitwise OR operation into a single output sequence of N bits. The nonlinear filter we used is the chaotic one dimensional tent map: $\mathcal{F}(m_n, e_n) = \mu$ for $m_n, m_n < \frac{1}{2}$ and $F(m_n, e_n)\mu(1-m_n)$ for $m_n \geq \frac{1}{2}$, with variable μ. We used $\mu = \mu_0 + (2-\mu_0)\beta_x e_n$, with $1 < \mu_0 \leq 2$ and $x = 1, 2$ indicating each map. In the chosen range of values of μ_0 and for any non rational number m_n, the transformation maps the interval $(0, 1)$ onto itself, generating white noise data with exponentially different trajectories for infinitesimally different input conditions. The advantage of this map is that it possesses a Lyapunov coefficient $\lambda = \log \mu$, which can be controlled from $\lambda \approx 0$ (chaos after an infinite number of steps) to $\log 2 = 0.69$ (chaos after a single iteration), allowing a fine control over the dynamics of exponential separation of nearby trajectories.
The values of the coefficients μ_0, β_1 and β_2 that guarantee a minimum bit error rate (BER) among the users are

calculated by training the algorithm from a dataset of PDS spectra of combined states P_{Alice} and P_{Bob} where:

- P_{Alice} is the spectrum measured by Alice.
- P_{Bob} is the spectrum measured by Bob.

We considered different communication scenario, in which Alice and Bob measure spectral differences $P_{Alice} - P_{Bob}$ with standard deviations $\sigma_{AB} = \sqrt{\langle (P_{Alice} - P_{Bob})^2 \rangle} \leq 8\%$. For each value of σ_{AB}, we generated a dataset of different different chaotic spectra with the following procedure. The chaotic spectrum P_{Alice} measured by Alice is generated from a random field field following Eqs. (1) of the main text with 1000 different frequencies. The spectrum measured by Bob is generated as $P_{Bob} = P_{Alice} + \Delta$, with the spectral difference Δ representing the uncertainty of Bob's measure.
In our analysis we considered uncertainty Δ with $\langle \Delta \rangle = 0$ and $\sqrt{\langle \Delta^2 \rangle} = \sigma_{AB}$, which represent statistical fluctuations of the source.
We find optimized values of μ_0, β_1 and β_2 against the following desired quantities:

- The minimal bit error rate (BER) between the bit sequences generated by Alice and Bob at their distal end.
- The average number of zeros N_z contained in the key generated by each user to be 50% of the key length. This is one of the various statistical requirements for the key to appear as random as possible [1].

These requirements are imposed by minimizing the norm of the following vectorial cost function C:

$$C(B_a, B_b, \mu_0, \beta_1, \beta_2) = (\mathrm{BER}, 0.5 - N_{za}, 0.5 - N_{zb}), \quad (8)$$

with B_x the bit sequence generated from the chaotic spectrum of each user: Alice (x=a), Bob (x=b), BER the bit error rate between B_a and B_b, N_{zx} the number of zeros in the key of each user. For each value of N and σ_{AB}, the minimization of C is carried by a Nelder-Mead algorithm [2].
Supplementary Figure 5 shows a typical training in the case of different N and $\sigma_{AB} = 8\%$. Supplementary Figure 5a plots the minimization of the cost function versus the number of bit sequences N_{ch} considered in the dataset for $N = 10$ (green), $N = 12$ (red) and $N = 16$ (blue). The function converges quickly, showing that very few spectra are necessary for training the AHB algorithm to generate optimized bit sequences. This result depends on the fact that we are generating bit sequences from chaotic spectra with universal probability density (see Fig. 2c of the main text). This implies that each spectrum has the same statistics and when a sufficiently large number of frequencies is considered, even a single spectrum is statistically representative. Supplementary Figure 5b shows the corresponding optimized values of μ_0, β_1 and β_2 for the three cases considered $N = 10, 12, 16$. They all converge to the same value independently from the N considered. The same result is observed by fixing N and changing σ_{AB}: optimized μ_0, β_1 and β_2 converge to the same value (not shown here for simplicity). This implies that the desired cost function C has a global minimum that allows to generate optimized bit sequences irrespective of the input conditions N and σ_{AB}.

SUPPLEMENTARY NOTE V

In the absence of active eavesdropping, the number of pulses N_p to be transmitted for generating an OTP key is:

$$N_p = \frac{L_m}{N_b \cdot P^{(ov)}}, \quad (9)$$

and equal to the ratio between the length of message L_m and the number of bits N_b extracted from each combined state $A_n \oplus B_{n'}$, multiplied by the probability $P^{(ov)}$ of observing a repeated state at the same time position in Alice and Bob's sequences. The maximum key generation throughput is observed when $P^{(ov)}$ is maximal.
Supplementary Figure 6 shows the probability $P^{(ov)}$ for different values of the users probabilities $P^{(Alice)}$ and $P^{(Bob)}$ to change state after each step. A maximum value of $P^{(ov)} = 10\%$ is obtained when $P^{(Alice)} = P^{(Bob)} = 0.25$. In this condition, by using standard detectors at 1024 bits and by extracting 10 bits per spectral sample, the protocol requires the transmission of $N_p = \frac{L_m}{1024}$ pulses, corresponding to $\approx 1/1000$ of the length of the message.
In the presence of active eavesdropping, the key generation throughput decreases due to the additional error states that the eavesdropper is introducing. According to the results of Supplementary Note II and Supplementary Figure 1, the throughput variation introduced by these attacks is in the range of a few percents.

SUPPLEMENTARY NOTE VI

Optical billiards are mathematical models for the study of chaos of classical and quantum waves propagating into microresonators. They study the particle dynamics of light following the motion of light rays inside the resonator, with elastic reflection at the boundary due to snell law [3, 4]. In the fingerprint resonators of Fig. 3 of the main text, the dynamics starts from an initial position $\mathbf{x}_0 = (x_0, y_0)$ and initial velocity vector $\mathbf{n} = (n_x, n_y) = (\cos\alpha, \sin\alpha)$, evolving the particle position $\mathbf{x}$ at $t + \Delta t$ by:

$$\begin{cases} x(t+\Delta t) = x_0 + n_x \Delta t, \\ y(t+\Delta t) = y_0 + n_y \Delta t, \end{cases} \quad (10)$$

as long as the particle does not collide with a scatterer. When this event happens at $\mathbf{x}_1 = (x_1, y_1)$, the particle is

reflected on the scatterer surface along the new velocity vector $\mathbf{n}_1$, and motion (10) starts again until the next collision.
A trajectory in the billiard evolution consists in the map $\mathcal{M} = (\mathbf{x}_0, \mathbf{x}_1, ..., \mathbf{x}_n)$ of the collision points at successive times until the particle escapes the billiard. As the particle motion is strongly chaotic, it is important not to use any numerical iterative solution for evaluating the scattering collision points $(\mathbf{x}_0, \mathbf{x}_1, ..., \mathbf{x}_n)$. In our study, we employ analytic solutions for calculating the intersection points between the trajectory of (10) and the scatterers, in order to estimate the properties of the chaotic dynamics as accurately as possible.
The calculation of the Lyapunov exponent follows from the algorithm detailed in [3]. The phase space of input conditions is represented by all the possible input vectors starting from an initial position $\mathbf{x}_0 = (0, y_0)$ displaced along the y axis, and directed along all the possible directions $\mathbf{n} = (\cos\alpha, \sin\alpha)$ with $\alpha \in [-\frac{\pi}{2}, \frac{\pi}{2}]$. For each input condition $(\mathbf{x}_0, \mathbf{n})$, we add the smallest spatial displacement ϵ representable at the computer and evolve the dynamics $(\mathbf{x}_0, \mathbf{n})$ and $(\mathbf{x}'_0 = x_0 + \epsilon, \mathbf{n})$, calculating the collisions maps $\mathcal{M} = (\mathbf{x}_0, \mathbf{x}_1, ..., \mathbf{x}_n)$ and $\mathcal{M}' = (\mathbf{x}'_0, \mathbf{x}'_1, ..., \mathbf{x}'_n)$ at successive iterations. We then evaluate the largest Lyapunov exponent by $\mu = \frac{1}{n} \log \frac{|\mathbf{x}_n - \mathbf{x}'_n|}{|\mathbf{x}_0 - \mathbf{x}'_0|}$. The average Lyapunov exponent is computed by average $\langle\mu\rangle$ over the space of different input positions $y_0 \in [-L, L]$, and angular orientations $\mathbf{n} = (\cos\alpha, \sin\alpha)$.

SUPPLEMENTARY NOTE VII

We consider the propagation of identical input conditions in billiard structures subjected to infinitesimal displacement of the scatterers positions $\mathbf{s}'_i \to \mathbf{s}_i + \epsilon$, being epsilon an infinitesimal quantity along an arbitrary direction of the space. The collision maps arising from the dynamics of same input condition launched in the original and displaced billiard are denoted as $\mathcal{M} = (\mathbf{x}_0, \mathbf{x}_1, ..., \mathbf{x}_n)$ and $\mathcal{M}' = (\mathbf{x}'_0, \mathbf{x}'_1, ..., \mathbf{x}'_n)$, respectively.
To demonstrate that the evolution represented by $\mathcal{M}$ and $\mathcal{M}'$ undergo exponential separation, we used mathematical induction. We begin by considering the dynamics at the first collision points $\mathbf{x}_0$ and $\mathbf{x}'_0$ starting from an identical input condition $(\mathbf{x}_{in}, \mathbf{n})$. Supplementary Figure 7a shows the geometrical calculation of the reflected trajectories, which impinge in the successive scatterer in the new collision points $\mathbf{x}_1$, $\mathbf{x}'_1$. Supplementary Figure 7b shows how the same construction can be obtained in an equivalent configuration by fixing the position of the scatterer to the original unmodified billiard, and displacing the input condition by the same infinitesimal transformation ϵ applied to the scatterer. The resulting distance among new collision points $d_{11'}$ can then expressed by:

$$d_{11'} = d_0 + \epsilon, \tag{11}$$

being d_0 the distance between the collision points as if they were originated from the unmodified billiard. As the latter is fully chaotic, the distance $d_0 = \epsilon e^{\mu}$ grows exponentially with respect to the original displacement ϵ. This implies that the distance $d_{11'} = \epsilon e^{\mu} + \epsilon \sim \epsilon e^{\mu}$ also grows exponentially.
We now consider the $n+1$-th collision $(\mathbf{x}_{n+1}, ..., \mathbf{x}'_{n+1})$, assuming that the distance $d_{n,n'} = |\mathbf{x}_n - \mathbf{x}'_n| \sim e^{\mu n}$ after the n-th collision grew exponentially. By repeating the same construction of Supplementary Figure 7, we obtain that the distance at the $n+1$ collision grows exponentially. By induction, this implies that the distance of the trajectories originated by an identical input condition in the original and modified billiards grow exponentially at the beginning and keeps increasing exponentially at every collision. For this family of transformations all trajectories will be affected and no old trajectory is observed in the modified billiard, as there will be at least one different reflection that will induce an exponentially diverging motion.
A similar result can be obtained by adding different scatterers to the fingerprint pattern, in the limit when each new scatterer is hit at least one time during the dynamics of one input condition in the unmodified structure. This is demonstrated as follows. Let us consider first the case of the addition of a single scatterer. Two identical input conditions in the original and modified resonator observe the same trajectory until one ray hits the new scatterer. From this point, one trajectory is reflected at a different angle. In a fully chaotic structure, this implies the creation of a trajectory that diverges exponentially form the first one. The addition of one scatterer generates a new family of exponentially diverging trajectories for all input conditions that, in the unmodified resonator, generated trajectories hitting the new scatter. When a sufficiently large number of scatterers is added and all input conditions hit at least a new scatter, all trajectories are modified exponentially.

SUPPLEMENTARY NOTE VIII

The integrated nature of the fingerprint chips enables ultrafast modulations with integrated light coupling. While the implementation of a high-dense device of this type goes beyond the scope of this paper, we here discuss a possible realization with the technology currently available.
The results of Fig. 4c show that the fingerprint chip, at every spatial point, generates uncorrelated data sequences from TE and TM polarized light. The use of different input position and light polarizations can be combined into the block scheme of Supplementary Figure 8. The initial stage is composed of a modified Mach-Zender, excited by an unpolarized source, or equivalently a combination of TE and TM polarized light sources. The Mach-Zender interferometer is composed by a $y-$junction fiber polarization splitter, followed by two ultrafast electro-optical (EO) modulators and a final y-junction for recombining the two polarization arms. The

output fiber is then connected to a $1 \times N$ optical switch matrix [5].
Both EO modulators and switch matrices can provide modulations up to hundreds of GHz and with hundreds of channels with the technology currently available [5], thus providing ultrafast selection of the input position on the fingerprint chip without the use of any mechanical component. The output light from the optical switch is then directly fed on to the fingerprint chip via multi-tapered channels couplers [6–8], providing on-chip couplings from end-to-end to the communication line. With this integrated structure, it is possible to achieve the generation of $4 \cdot N^2$ different spectra at each communication.

SUPPLEMENTARY REFERENCES

[1] L. E. Bassham, *et al.*, *SP 800-22 Rev. 1a. A Statistical Test Suite for Random and Pseudorandom Number Generators for Cryptographic Applications*, Tech. Rep. (Gaithersburg, MD, United States, 2010).

[2] F. Gao and L. Han, Computational Optimization and Applications **51**, 259 (2012).

[3] E. Ott, *Chaos in Dynamical Systems* (Cambridge University Press, 2002).

[4] N. Chernov and R. Markarian, *Chaotic billiards* (American Mathematical Society, 2006).

[5] R. Stabile, A. Albores-Mejia, A. Rohit, and K. A. Williams, Microsystems & Nanoengineering **2**, 15042 (2016), review Article.

[6] F. E. Doany, *et al.*, Journal of Lightwave Technology **29**, 475 (2010).

[7] V. R. Almeida, R. R. Panepucci, and M. Lipson, Optics letters **28**, 1302 (2003).

[8] Y. Vlasov, W. M. Green, and F. Xia, Nature photonics **2**, 242 (2008).
